# A comprehensive review of IgG4-related pancreatitis: pathogenesis, diagnosis, and therapeutic advances

**DOI:** 10.3389/fimmu.2025.1590902

**Published:** 2025-06-17

**Authors:** Siqin Wu, Xiaowen Qin, Zhenjing Jin, Lanlan Yang

**Affiliations:** The Second Hospital of Jilin University, Changchun, Jilin, China

**Keywords:** IgG4-related pancreatitis, IgG4-related disease, diagnosis, treatment, pathological mechanism

## Abstract

IgG4-related pancreatitis (IRP) is a form of chronic pancreatitis characterized by the infiltration of IgG4-positive plasma cells, representing a pancreatic manifestation of IgG4-related disease (IgG4-RD). In recent years, with the growing understanding of IgG4-RD, the incidence of IRP has shown an increasing trend. This article provides a comprehensive review of the latest advancements in the epidemiology, etiology, pathogenesis, clinical manifestations, diagnosis, differential diagnosis, and treatment of IRP.

## 1 Background

IgG4-related disease (IgG4-RD) is a chronic inflammatory disorder mediated by autoimmune abnormalities, often accompanied by fibroproliferation, which can affect multiple glands, organs, and systems throughout the body. The digestive system is most commonly involved in the liver, bile ducts, and pancreas. Autoimmune pancreatitis is primarily classified into two types: type 1 and type 2. IRP is the pancreatic manifestation of IgG4-RD, mainly corresponding to type 1 autoimmune pancreatitis (AIP-I). This disease is typically characterized by pancreatic enlargement, elevated serum IgG4 levels, and extensive infiltration of IgG4-positive plasma cells in tissues. Type 2 autoimmune pancreatitis often occurs in the context of inflammatory bowel disease, with histopathology frequently demonstrating granulocytic epithelial lesions and no significant association with IgG4-positive plasma cell or lymphocyte infiltration (1). Currently, most clinicians have a limited understanding of IRP, often misdiagnosing it as chronic pancreatitis, pancreatic cysts, or pancreatic malignancies. Therefore, this article aims to provide a systematic review of the current state of IgG4-IRP regarding its etiology, pathogenesis, clinical manifestations, imaging characteristics, and treatment options. It is intended to provide a reference for clinical diagnosis and treatment practices of IRP, assist clinicians in constructing a systematic diagnostic thinking framework, enhance comprehensive knowledge and accurate diagnostic capabilities for this disease, and avoid misdiagnosis that could delay treatment and affect patients’ quality of life.

## 2 Epidemiology

The epidemiological characteristics of IRP reveal variations in incidence based on geography, age, and gender, and it frequently coexists with other IgG4-RD. The incidence of IgG4-related pancreatitis differs significantly across regions, largely due to disparities in diagnostic awareness, clinical expertise, and diagnostic criteria. According to epidemiological data from Japan, the incidence of AIP-1 is notably higher there. Between 2007 and 2016, the incidence of type 1 AIP increased from 0.8 to 3.1 per 100,000 individuals, with an overall prevalence of 10.1 per 100,000 people (2). In contrast, the incidence of AIP-1 in Europe and the United States is relatively lower, approximately 0.8 to 1.2 per 100,000 person-years. However, with advancements in diagnostic techniques and increased awareness worldwide, the detection rate of this disease has been gradually rising.

IRP primarily affects middle-aged and elderly populations, with the peak onset age between 50 and 70 years. Male patients significantly outnumber females, with a male-to-female ratio of about 2:1 to 3:1. This gender disparity may be attributed to hormonal influences or sex-based differences in immune responses (3).

## 3 Etiology and pathogenesis

The exact etiology of IgG4-related pancreatitis (IRP) remains incompletely understood. Current clinical evidence suggests potential associations with infections, environmental exposures, immune dysregulation, gut microbiota disturbances, genetic predisposition, and familial susceptibility. The disease may result from either a single causative factor or, more likely, a combination of multiple factors (4).

IRP is an immune-mediated disorder characterized primarily by organomegaly and fibrosis-induced dysfunction. The key immune cell subsets implicated in its pathogenesis include:T follicular helper 2 cells (Tfh2)、T follicular helper type 1 cells (Tfh1)、Regulatory T cells (Tregs)、Cytotoxic CD4+ T cells (CD4+ CTLs)and so on (5, 6). In this review, we provide a concise overview of the common immunological mechanisms underlying IgG4-related disease (IgG4-RD) and IgG4-related pancreatitis (IRP).

### 3.1 Biological functions of IgG4

The infiltration of IgG4-positive plasma cells in the pancreas and other affected organs is a pathognomonic feature of IgG4-RD, leading to characteristic chronic inflammatory and fibrotic changes in IRP. IgG4, a subclass of immunoglobulin G, exhibits unique biological properties including immunological inertness and Fab-arm exchange capability. Through Fc-Fc interactions, IgG4 demonstrates dual functionality: (1) serving as a monovalent antibody with anti-inflammatory effects, while (2) forming immune complexes that activate the alternative complement pathway (5, 7). IgG4-positive plasma cells infiltrate small veins, leading to occlusive phlebitis, one of the characteristic pathologic manifestations of IgG4-RD (8).

### 3.2 Role of cytokines and immune cell subsets

*In vitro* functional analyses demonstrate that Tfh2 cells possess the capacity to induce naive B cell differentiation into IgG4-secreting plasma cells. Notably, Tfh2 cells derived from IgG4-related disease (IgG4-RD) patients exhibit significantly enhanced potency in driving this IgG4+ plasma cell differentiation compared to those from healthy controls (9, 10).

Transforming growth factor-β (TGF-β) and interleukin-10 (IL-10) mediate fibroblast activation, while follicular helper T cells (Tfh) promote B cell activation and IgG4 class switching via secretion of IL-4 and IL-21. Treg contribute to IgG4 production and fibrosis by secreting TGF-β, which also exhibits anti-inflammatory properties, suggesting a potential close association between TGF-β and fibrogenesis (11). Dysfunctional Treg cells, hyperactivated Tfh cells, and abnormal B cell proliferation collectively result in immune dysregulation, perpetuating chronic inflammation and progressive fibrosis (12).

### 3.3 Formation of tertiary lymphoid structures in IgG4-RD

Researchers such as Aoyagi have observed the presence of tertiary lymphoid structures (TLS) in the lesional tissues of patients with IgG4-related disease (IgG4-RD). These structures, which resemble secondary lymphoid organs, serve as critical sites for localized immune responses. TLSs are populated by various immune cells, including T cells, B cells, and macrophages, all of which play pivotal roles in the pathological processes of IgG4-RD. Further studies have revealed that B cells within TLSs, aided by follicular helper T cells (Tfh), may undergo class-switch recombination to produce substantial amounts of IgG4 antibodies. This finding suggests that B cells within TLSs are likely closely associated with localized inflammatory responses and fibrotic processes (13, 14).

## 4 Diagnosis of IgG4-SC

In the early stages, the diagnostic criteria for IRP were not standardized internationally. Variability in regional and population differences led to limitations in clinical application. For instance, the Japanese Comprehensive Diagnostic Criteria (15), the ACR/EULAR Classification Criteria (16), and the Chinese Expert Consensus (17) each had their own frameworks. Additionally, IRP often involves multiple organs and systems, and the understanding and diagnostic criteria varied across specialties such as gastroenterology, rheumatology, and radiology. This lack of interdisciplinary collaboration frequently resulted in misdiagnosis or delayed diagnosis. In 2011, the International Consensus Diagnostic Criteria (ICDC) for autoimmune pancreatitis were established by the International Association of Pancreatology. These criteria proposed a comprehensive clinical diagnosis of IRP based on five key aspects: clinical manifestations, serological findings, extrapancreatic involvement, pancreatic histopathology, and response to steroid therapy (collectively known as the HISORt criteria) (15, 18), It is currently one of the most widely used diagnostic criteria. The following section provides a systematic review of the current application of clinical diagnosis and differential diagnosis for IRP.

### 4.1 Clinical manifestations

The clinical manifestations of IgG4-related pancreatitis are diverse. Common symptoms include abdominal pain, bloating, weight loss, and dyspepsia. A minority of patients may exhibit signs of impaired pancreatic exocrine function, such as steatorrhea, postprandial distension, and blood glucose fluctuations. Additionally, jaundice and pruritus are frequent symptoms when IRP involves the extra-pancreatic bile ducts (19, 20). However, the majority of patients exhibit an insidious onset with no obvious symptoms, and the condition is often incidentally detected through imaging studies revealing diffuse pancreatic enlargement. The disease course is characterized by a tendency for recurrent episodes.

To date, the international diagnostic consensus for IRP has included extrapancreatic manifestations as one of the clinical diagnostic criteria, emphasizing their importance in the overall assessment of disease burden. Additionally, the involvement of extrapancreatic organs can assist clinicians in differentiating between type 1 and type 2 autoimmune pancreatitis (15). Studies report that 61% to 95% of patients may exhibit IgG4-RD in other organs, either synchronously or metachronously. These include IgG4-related cholangitis, tubulointerstitial nephritis, pulmonary diseases, orbital pseudotumors, sialadenitis, retroperitoneal fibrosis, and other (21–24). The signs and symptoms of IgG4-RD often arise directly from the enlargement of affected organs, which can lead to compression of adjacent structures. Examples include ureteral obstruction in cases of retroperitoneal involvement and optic nerve compression in orbital disease (25).

### 4.2 Serological testing

Elevated serum IgG4 levels are one of the key diagnostic indicators for IRP. However, the specificity of elevated serum IgG4 levels is relatively low, as other conditions such as pancreatic cancer and chronic pancreatitis may also present with increased IgG4 levels. Notably, significantly elevated serum IgG4 levels can also be observed in various other immune-related disorders, including chronic malignancies, long-term infections, and immune-mediated vasculitides (26). In 2011, the Japanese IgG4-RD Research Association proposed a standardized serum IgG4 cutoff value of >1.35 g/L, which better aligns with clinical practice. Generally, a higher proportion of Asian IRP patients exhibit elevated serum IgG4 levels, while the prevalence is lower among patients in Europe and the United States (27). A case report from Sweden highlighted that a small subset of patients with AIP-Iinitially tested negative for elevated serum IgG4 levels, with levels rising only in the later stages of the disease. The report suggested that a serum IgG4 level exceeding four times the upper limit of normal should be considered clinically significant for diagnostic purposes (28). However, only a minority of patients meet this criterion. The sensitivity and specificity of elevated serum IgG4 levels are insufficient to differentiate IgG4-RD from other autoimmune conditions, such as distinguishing IgG4-related cholangitis from primary biliary cholangitis or IgG4-related pancreatitis from type 2 autoimmune pancreatitis (AIP-II) associated with inflammatory bowel disease (29).

A small proportion of IRP patients exhibit normal or mildly elevated serum IgG4 levels, which can lead to missed or incorrect diagnoses, particularly in the early stages of the disease or when only localized organs are involved. This delay can hinder timely treatment. Furthermore, the correlation between serum IgG4 levels and disease activity remains unclear, making it an unreliable indicator of disease progression or treatment efficacy. A retrospective study from Japan suggested that elevated serum IgG4 levels might predict poor outcomes in untreated IgG4-RD patients. These patients often had fewer affected organs and lower IgG4-RD response indices. However, this conclusion requires validation through larger-scale, multicenter prospective studies (30).

Recent studies have revealed that serum CA19–9 levels are frequently elevated to varying degrees in pancreatic cancer, chronic pancreatitis, and IRF (immune-related fibrosis). The use of a single clinical serological marker is insufficient for the accurate differential diagnosis of pancreatic diseases. Therefore, a combination of serological markers, including IgG4, CA19-9, and CEA, is often required to enhance diagnostic accuracy and reduce the rate of misdiagnosis (31, 32). The sensitivity and specificity of serum IgG4 levels remain subjects for further investigation. Therefore, elevated serum IgG4 levels are not a mandatory criterion for diagnosing IRP and must be interpreted in conjunction with other diagnostic findings for a comprehensive assessment.

### 4.3 Imaging tests

Imaging studies play a crucial role in the diagnosis of IRP. Conventional imaging modalities, such as computed tomography (CT), magnetic resonance imaging (MRI), and ultrasonography, are commonly used. CT is the preferred imaging method for diagnosing AIP and holds significant value in differentiating AIP from pancreatic cancer (17). Conventional imaging examinations often have difficulty distinguishing between the inflammatory phase and the fibrosis phase, which affects the formulation of treatment strategies. Common imaging findings of IRF include diffuse or focal enlargement of the pancreas (resembling a ‘sausage’), accompanied by delayed enhancement in the venous phase, and the feather-like structures of the pancreas may be hazy or even disappear (33), Magnetic resonance cholangiopancreatography (MRCP) or CT-guided cholangiopancreatography may reveal segmental narrowing or dilation of the main pancreatic duct (29), The value of MRCP in assessing the degree of stenosis and dilation of the main pancreatic duct requires further investigation. The bile ducts are the most frequently involved extrapancreatic organs in IRP. Infiltration of IgG4-positive plasma cells into the bile duct walls can lead to varying degrees of stenosis and dilation in the pancreatic duct and intrahepatic or extrahepatic bile ducts. The evaluation of pancreatic duct (PD) and common bile duct (CBD) diameter and wall thickness is considered one of the key radiological diagnostic features of AIP (34–36).In a multivariate regression analysis, we identified a significant association between the thickness of PD and CBD and the diagnosis of AIP-I (37). Diffuse pancreatic enlargement may serve as a significant predictor of late-stage IRP recurrence (38). However, during disease progression, persistent tissue fibrosis in IRP frequently leads to the formation of chronic inflammatory masses. When these present as localized inflammatory lesions, they are often misdiagnosed as pancreatic malignancies, consequently resulting in unnecessary surgical interventions.

Conventional Doppler endoscopic ultrasound (EUS) derives its imaging signals from motion artifacts and blood flow, serving as a relatively reliable and effective diagnostic modality for hepatopancreaticobiliary diseases with high spatial resolution. In patients with AIP, EUS imaging typically demonstrates diffuse hypoechoic pancreatic enlargement. However, relying solely on EUS imaging characteristics poses certain challenges for clinical differential diagnosis (29). DFI-EUS (Doppler Flow Imaging Endoscopic Ultrasound) constructs images by separating blood flow signals from superimposed tissue motion artifacts, while preserving signals from low-flow components. This enables the definition and visualization of extremely fine vasculature. A retrospective study demonstrated that DFI-EUS exhibits superior sensitivity in vascular detection compared to conventional EUS, particularly showing higher diagnostic sensitivity for pancreatic cancer. The vascular pattern assessment by DFI-EUS can be utilized for preliminary differential diagnosis of pancreatic masses, autoimmune pancreatitis, and other pancreatic diseases (39).

### 4.4 Pathological diagnosis

Characteristic histopathological alterations serve as crucial diagnostic criteria for AIP, underscoring the necessity of obtaining adequate tissue samples for accurate pathological evaluation. Traditional surgical approaches for pancreatic tissue acquisition carry significant risks, including potential complications such as pancreatic hemorrhage, pancreatic fistula, and pancreatitis, which may become life-threatening in severe cases. Moreover, key pathological features like storiform fibrosis and obliterative phlebitis might be missed due to limited biopsy sampling or suboptimal sampling sites. Additionally, pathological manifestations may vary across different organs or even within different regions of the same organ - for instance, head and neck lesions may lack typical fibrotic features. Given these challenges, endoscopic ultrasound-guided fine-needle biopsy (EUS-FNB) is currently recommended as the preferred diagnostic approach (17, 40).

Endoscopic ultrasonography (EUS) serves not only as an imaging diagnostic tool for IRP but also as a method for obtaining tissue samples from affected pancreatic regions. Compared to traditional pancreatic surgical sampling, EUS-guided fine-needle aspiration (EUS-FNA) or biopsy (EUS-FNB) offers a safer alternative (29, 37, 41). A meta-analysis revealed that histopathological results obtained through endoscopic ultrasound-guided fine-needle aspiration (EUS-FNA) provided diagnostic significance in 54.7% of cases, while the diagnostic rate for histological examination via FNA was 21.9% (42). A multicenter randomized prospective study conducted by Sung et al. divided 100 patients into two groups based on different sampling sites, targeting the central and peripheral zones of pancreatic masses larger than 3 cm. The results demonstrated no significant difference between the two groups when using endoscopic ultrasound-guided fine-needle biopsy (EUS-FNB). Multipoint sampling was shown to improve biopsy success rates and could also be utilized to identify specific subtypes of autoimmune pancreatitis (AIP) and to differentiate between focal manifestations of IRP and pancreatic malignancies (43, 44). The key differential characteristics between Type 1 and Type 2 autoimmune pancreatitis (AIP-1 and AIP-2) are summarized in **Table 1** (1, 45, 46).

**Table 1:** Differential diagnosis of AIP-I and AIP-II.

Category	AIP-I	AIP-II
Epidemiology	Asian > Caucasian	Caucasian > Asian
Gender	Older	Younger
Age	Male > > female	Male≈ female
Serum levels of IgG4	Elevated	Normal
Predominant Infiltrating Cells	Lymphocytes and plasma cells	Neutrophils
Pathology	Lymphoplasmacytic sclerosing pancreatitis, often meeting IgG4-RD criteria	Idiopathic duct-centric pancreatitis with granulocytic epithelial lesions
Common Disease Context	IgG4-RD	Inflammatory bowel disease
Disease Characteristics	Multisystem involvement	Primarily localized to the pancreas

## 5 Medical treatment of IgG4-associated pancreatitis

### 5.1 Glucocorticoids therapy

In the absence of contraindications to glucocorticoids (GCs), corticosteroids are the first-line treatment for all newly diagnosed IRP patients in the active phase of the disease. Most patients respond well to steroid therapy, with significant improvements in symptoms and imaging findings observed within a short period. Once IRP is diagnosed, prompt initiation of aggressive pharmacological treatment is recommended. The commonly used starting dose is prednisone at 0.6–1.0 mg/kg/day, maintained for 2–4 weeks, followed by a gradual taper. The dose is reduced by 5 mg every 2 weeks, with the goal of discontinuing the medication within 3–6 months to achieve clinical remission. Alternatively, long-term maintenance therapy may be considered to delay or prevent irreversible organ damage (47–49).

Even if patients with IRP respond well to initial glucocorticoid (GC) therapy, the therapeutic efficacy of GCs can vary during the course of treatment. In many cases, tapering and discontinuation of corticosteroids are associated with a high risk of disease relapse (47, 49–51). A Kaplan-Meier multivariate analysis confirmed that glucocorticoid administration reduces the risk of progression to chronic pancreatitis (CP) in patients with autoimmune pancreatitis (AIP) presenting with pancreatic head swelling. It also decreases the incidence of severe pancreatic tissue calcification, suggesting that glucocorticoids may prevent or delay the progression of AIP toward CP (52). A retrospective study from Japan, which included data from 510 patients, concluded that typical AIP patients should receive maintenance steroid therapy (MST) at an appropriate dose, specifically 5 mg/day of prednisolone, for 2–3 years. This regimen can reduce the disease relapse rate to below 30% while minimizing the side effects associated with glucocorticoids (38), However, the efficacy of maintenance steroid therapy (MST) in reducing the risk of relapse in patients with autoimmune pancreatitis (AIP) remains controversial. Larger-scale retrospective studies or long-term follow-up trials are needed to further validate its effectiveness.

### 5.2 Relapse after glucocorticoid therapy

Relapse in IgG4-related disease (IgG4-RD) can be categorized into two types: clinical relapse and serological relapse (53) (**Table 2**). An elevation in serum IgG4 levels alone is not considered indicative of relapse. The IgG4-RD Responder Index (IgG4-RD RI), proposed by the International IgG4-RD Study Group, serves as a standardized tool for assessing disease activity and treatment response in IgG4-related disease (IgG4-RD). The IgG4-RD RI typically comprises three components: organ involvement score, serological score, and symptom score (54). The European guidelines propose that the assessment of disease activity in IgG4-related disease (IgG4-RD) should primarily rely on a comprehensive evaluation incorporating physical examination findings, laboratory test results, histopathological features, and imaging studies (29).

**Table 2:** Types of relapse in IgG4-RD.

Type	Definition
Clinical Relapse	The appearance or worsening of any organ dysfunction, swelling, or space-occupying lesions detected on physical or radiological examination, with or without elevated serum IgG4 levels.
Serological Relapse	Stable clinical symptoms with an increase in serum IgG4 levels, resulting in an IgG4-RD Responder Index (IgG4-RDRI) score exceeding 1 point.

Post-treatment relapse has been reported in approximately 10-20% of IRP patients (41).A univariate study from Sweden suggested that age and the number of organs involved may be two significant factors influencing relapse after glucocorticoid therapy in autoimmune pancreatitis (AIP). However, these findings have certain limitations (1). Other potential high-risk factors may include a history of relapse following steroid therapy, bile duct involvement, diffuse pancreatic enlargement, and elevated serum IgG4 levels (29, 38, 55). Wallace et al. also found that baseline serum IgG4 and IgE concentrations, as well as eosinophil levels, are significant predictors of relapse (56).

Based on the evidence available to date, although nearly all IgG4-RD patients show significant initial improvement with glucocorticoid therapy, some may experience clinical or serological relapse during dose reduction or after discontinuation of steroids. A prospective cohort study conducted at Peking Union Medical College Hospital demonstrated that the initial efficacy of glucocorticoid monotherapy was comparable to that of combination therapy, but significant differences emerged post-treatment. Prednisone combined with cyclophosphamide (CYC) significantly reduced relapse rates. Patients who relapsed on monotherapy showed a favorable response to immunosuppressive therapy. The 1-year remission rates were 59.6% in the glucocorticoid monotherapy group and 88.0% in the glucocorticoid plus cyclophosphamide group (57). There is currently no definitive evidence demonstrating that the combination of glucocorticoid-sparing immunomodulators (IMs) with glucocorticoid therapy is significantly superior to glucocorticoid monotherapy (58).

### 5.3 Immunosuppressant therapy

Immunosuppressants, also known as disease-modifying antirheumatic drugs (DMARDs), can serve as second-line treatment options for IgG4-RD patients who are steroid-dependent or experience relapse after initial therapy. Common immunosuppressants include azathioprine (AZA), cyclophosphamide (CTX), mycophenolate mofetil (MMF), leflunomide (LEF), 6-mercaptopurine (6-MP), methotrexate (MTX), and tacrolimus (59), When combined with glucocorticoid therapy, these drugs can reduce the required dose of steroids, lower the risk of disease relapse, and mitigate the side effects associated with steroid treatment (e.g., impaired glucose tolerance, hypertension, osteoporosis, etc.). Combination therapy can be used for both initial induction of remission and re-induction during disease relapse (50). According to current research evidence, there is no universally recommended dose for long-term glucocorticoid maintenance therapy in international guidelines or expert consensus. Long-term low-dose steroid therapy may increase the risk of disease relapse, while long-term high-dose steroid therapy can elevate the incidence of steroid-related side effects and even potentially trigger certain rheumatic or immune-related conditions. Baseline high disease activity (IgG4-RDRI > 9) and the absence of rituximab (RTX) maintenance therapy are also risk factors for relapse. Additionally, a small number of patients may develop hypogammaglobulinemia and infections (60).

A retrospective study collected clinical data from IgG4-RD patients treated with Group I (GCs + LEF) and Group II (GCs + MMF) combination therapies. Statistical analysis revealed that both groups exhibited excellent short-term treatment outcomes, with nearly 100% of patients achieving a therapeutic response. During long-term follow-up, Group II demonstrated a higher overall remission rate, longer duration of disease remission, and a lower incidence of adverse events compared to Group I (61).

### 5.4 Targeted drug therapy

Rituximab (RTX) is an anti-CD20 monoclonal antibody that depletes B cells, reducing the infiltration of IgG4-positive plasma cells and thereby controlling disease activity and progression. In recent years, RTX has demonstrated promising efficacy in the treatment of IgG4-related pancreatitis. For refractory or relapsing patients, monoclonal antibody therapy represents a promising treatment option. A meta-analysis by Omar et al. showed that the RTX maintenance therapy group had the lowest disease relapse rate compared to all other treatment groups (GCs alone, GCs combined with non-biologic DMARDs, and RTX induction therapy) (62). Rituximab (RTX) therapy is highly effective for inducing and maintaining remission in patients with autoimmune pancreatitis (AIP), whether accompanied by IgG4-related sclerosing cholangitis or not. It is associated with a low risk of disease relapse and treatment-related side effects (58). A recent retrospective cohort study in the United States involving 60 IgG4-RD patients treated with rituximab (RTX) reported a relapse rate of 37% over an average follow-up period of 8 months. The study also revealed that B-cell reconstitution is one of the key mechanisms underlying late relapse in IgG4-RD patients (56). A nationwide study in France involving 33 patients found that the relapse rate in the RTX-treated cohort was 42% over an average follow-up period of 25 months (63).

Obexelimab is a CD19xFcγRIIB bispecific antibody whose mechanism of action is primarily based on its dual-targeting design. It simultaneously targets CD19 and F cγ RIIB, thereby modulating B-cell function and suppressing excessive immune responses. A single-center phase 2 pilot trial in the United States reported that some patients exhibited significant symptom improvement and organ function recovery after treatment, such as remission of pancreatic, salivary gland, or biliary lesions. The therapy also markedly reduced serum IgG4 levels, indicating its inhibitory effect on abnormal immune activity (64).

### 5.5 Surgical treatment

The application of surgical intervention in IgG4-related pancreatitis is relatively limited and is primarily reserved for the following scenarios:

- Unclear Diagnosis: When imaging and serological tests fail to provide a definitive diagnosis and malignancy is suspected, surgical resection may be performed to establish a diagnosis.
- Management of Complications: Complications such as obstructive jaundice due to biliary strictures or pancreatic pseudocysts may require biliary drainage or surgical intervention (48).
- Refractory Cases: Surgery may be considered as a last resort for refractory cases that do not respond to medical therapy. However, surgical intervention is not the first-line treatment for IgG4-related pancreatitis due to the significant trauma associated with the procedure and the need for long-term follow-up (29).

When AIP-I involves the bile ducts, causing obstructive jaundice due to biliary drainage obstruction, endoscopic nasobiliary drainage (ENBD) can not only alleviate clinical symptoms but also enable real-time cholangiography to assess the efficacy of glucocorticoid therapy, thereby reducing the need for repeated endoscopic retrograde cholangiopancreatography (ERCP) procedures (65).

## 6 Summary and outlook

IRP is the pancreatic manifestation of IgG4-RD. The diagnosis of IRP primarily relies on a comprehensive assessment of clinical features, including serological findings, extrapancreatic involvement, imaging characteristics, histopathological results, and response to glucocorticoid therapy. The clinical manifestations of IRP mainly include digestive symptoms such as abdominal pain, bloating, and weight loss, which are often nonspecific. Some patients may develop jaundice due to biliary involvement. Serologically, elevated IgG4 levels are a hallmark, but their specificity and sensitivity are relatively low. Focal mass-type IRP can be challenging to distinguish from pancreatic malignancies on CT or MRI imaging. EUS is increasingly being adopted in clinical practice. Although glucocorticoid therapy carries risks of steroid dependence and relapse, it remains the first-line induction treatment and cornerstone of therapy for IgG4-RD with multi-organ involvement. Glucocorticoids show significant efficacy in the early stages of treatment. For patients with inadequate response to glucocorticoid monotherapy or relapse during maintenance therapy, combination therapy with immunosuppressants or targeted biologics may be considered. When medical therapy is ineffective, surgical intervention can be selectively employed based on the patient’s condition, aiming to develop a more precise and personalized treatment strategy.

Future research should focus on exploring novel serological biomarkers for IRP through multi-omics approaches and B-cell subset analysis. By integrating genomic, proteomic, and metabolomic technologies, we aim to identify disease-specific markers associated with disease activity (e.g., cytokines such as IL-4, IL-10, and TGF-β, or other immune markers). Experimental studies should investigate the correlation between peripheral blood B-cell subsets/plasmablast dynamics and disease activity. Large-scale cohort studies will be essential to validate these potential biomarkers or novel technologies, ultimately facilitating clinical translation to delay or prevent disease recurrence and progression.

With in-depth research into the pathogenesis of IRP, targeting pathways involving T follicular helper (Tfh) cells or IL-4, as well as novel pathophysiological mechanisms, we can further explore the potential of combining biologic targeted therapies with glucocorticoids or other immunosuppressants. More targeted therapeutic agents are expected to emerge, improving patient prognosis. Long-term follow-up will help assess disease relapse rates and drug efficacy. We eagerly anticipate randomized controlled trials to confirm the promising preliminary findings reported in case series and small cohort studies. The efficacy and safety of these drugs require further validation through larger-scale, multicenter randomized controlled trials, such as phase III clinical trials.

In summary, early diagnosis of IRP and the optimization of personalized treatment strategies will be key focuses of future research. Additionally, long-term follow-up and disease management mechanisms need further refinement.
